# Early metabolic response of breast cancer to neoadjuvant endocrine therapy: comparison to morphological and pathological response

**DOI:** 10.1186/s40644-020-0287-4

**Published:** 2020-01-28

**Authors:** Sarah Boughdad, Laurence Champion, Veronique Becette, Pascal Cherel, Emmanuelle Fourme, Jerome Lemonnier, Florence Lerebours, Jean-Louis Alberini

**Affiliations:** 1 Department of Nuclear Medicine, Institut Curie-Saint-Cloud, 92210 Saint-Cloud, France; 2 Department of Pathology, Institut Curie, Saint-Cloud, France; 3 Department of Radiology, Institut Curie, Saint-Cloud, France; 4 Department of Statistics, Institut Curie, Saint-Cloud, France; 5 UNICANCER, UCBG, Paris, France; 6 Department of Medical Oncology, Institut Curie, Saint-Cloud, France; 7 Université Versailles Saint-Quentin, Paris-Saclay, Saint-Quentin-en-Yvelines, France

**Keywords:** Breast cancer, Neoadjuvant endocrine therapy, Estrogen receptor-positive / HER2-negative, FDG, PET/CT, Metabolic response, Morphological response, Pathological response

## Abstract

**Background:**

Neoadjuvant endocrine therapy (NET) has shown efficacy in terms of clinical response and surgical outcome in postmenopausal patients with estrogen receptor-positive / HER2-negative breast cancer (ER+/HER2- BC) but monitoring of tumor response is challenging. The aim of the present study was to investigate the value of an early metabolic response compared to morphological and pathological responses in this population.

**Methods:**

This was an ancillary study of CARMINA 02, a phase II clinical trial evaluating side-by-side the efficacy of 4 to 6 months of anastrozole or fulvestrant. Positron Emission Tomography/Computed Tomography using 2-deoxy-2-[^18^F]fluoro-D-glucose (FDG-PET/CT) scans were performed at baseline (M0), early after 1 month of treatment (M1) and pre-operatively in 11 patients (74.2 yo ± 3.6). Patients were classified as early “metabolic responders” (mR) when the decrease of SUVmax was higher than 40%, and “metabolic non-responders” (mNR) otherwise. Early metabolic response was compared to morphological response (palpation, US and MRI), variation of Ki-67 index, pathological response according to the Sataloff classification and also to Preoperative Endocrine Prognostic Index (PEPI) score. It was also correlated with overall survival (OS) and recurrence-free survival (RFS).

**Results:**

Tumor size measured on US and on MRI was smaller in mR than mNR, with the highest statistically significant difference at M1 (*p* = 0.01 and 7.1 × 10^− 5^, respectively). No statistically significant difference in the variation of tumor size between M0 and M1 assessed on US or MRI was observed between mR and mNR. mR had a better clinical response: no progressive disease in mR vs 2 in mNR and 2 partial response in mR vs 1 partial response in mNR. One patient with a pre-operative complete metabolic response had the best pathological response. Pathological response did not show any statistically significant difference between mR and mNR. mR had better OS and RFS (Kaplan-Meier *p* = 0.08 and 0.06, respectively). All cancer-related events occurred in mNR: 3 patients died, 2 of them from progressive disease.

**Conclusions:**

FDG-PET/CT imaging could become a “surrogate marker” to monitor tumor response, especially as NET is a valuable treatment option in postmenopausal women with ER+/HER2- BC.

## Introduction

Neoadjuvant systemic therapies have been developed to achieve “tumor shrinkage” in locally advanced breast cancer in inoperable patients or to avoid radical mastectomy in patients with a tumor too large for primary breast-conserving surgery (BCS) [1] and to monitor tumor response. Nearly 70% of breast cancers (BC) express hormone receptors and estrogen receptor-positive (ER+) and are less responsive to chemotherapy than ER-negative BC [2]. Neoadjuvant endocrine therapy (NET) is a recognized option of treatment for postmenopausal women with ER+/HER2- BC [3]. Pathological complete response (pCR) is the “gold standard” for evaluation of tumor response to neoadjuvant chemotherapy (NCT), as it is correlated with prognosis, although differences are observed among breast cancer subtypes [2]. However, pCR is uncommon after NET in ER+/HER2- BC [4] and is therefore not a suitable primary endpoint in NET clinical trials. Monitoring response to NET is challenging and, despite a poor reproducibility, clinical response is used as a primary endpoint in most clinical trials [5]. Monitoring of Ki67 index, a proliferation biomarker, has been increasingly used in NET clinical trials [5].

Imaging techniques such as breast ultrasound (US) [6] or MRI [7] have shown interesting results in neoadjuvant setting, but none of these techniques has been shown to be superior to clinical response in NET setting. Positron Emission Tomography/ Computed Tomography using 2-deoxy-2-[^18^F]fluoro-D-glucose (FDG-PET/CT) could therefore be a valuable tool to monitor in vivo changes in tumor glucose metabolism. FDG-PET/CT has shown efficacy for BC staging [8, 9], monitoring of tumor response to NCT [10] and detection of recurrence [11]. CARMINA 02 (NCT00629616, [12]) is a French phase II multicenter, randomized neoadjuvant trial evaluating side-by-side the efficacy of anastrozole and fulvestrant in postmenopausal patients with non-metastatic ER+/HER2- BC. The aim of the present study was to investigate the value of an early metabolic response on FDG-PET/CT after 1 month of NET compared to morphological response assessed by palpation and imaging with US and MRI, variation of Ki-67 index and pathological response. The predictive value of early metabolic response for prognosis, survival and patient management, in terms of BCS rate and adjuvant therapy, were defined as secondary objectives.

## Material and methods

### Study design

**Fig. 1:**
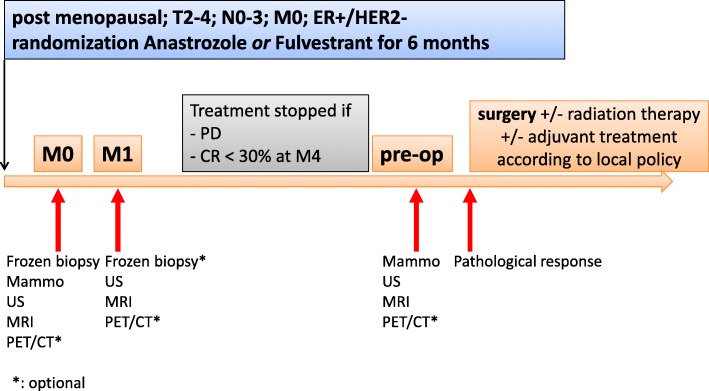
CARMINA 02 trial flow chart

The present study was an ancillary study of CARMINA 02 prospective trial ([12], Fig. 1). The primary endpoint of this trial was the clinical response rate according to RECIST 1.0 criteria [12] assessed after 4 to 6 months in each treatment arm. Secondary endpoints were tumor response on US and MRI according to RECIST 1.0 criteria, baseline and on-treatment Ki-67 index, BCS rate, pathological response assessed by the Sataloff classification, with partial pathological response defined as TB and pCR as TA [13], and survival parameters, with overall survival (OS), recurrence-free survival (RFS) and PEPI score (Preoperative Endocrine Prognostic Index, [14]). Metabolic response based on FDG-PET/CT was an optional secondary endpoint in CARMINA 02 trial [15] and is the object of the present study. Post-menopausal patients with ER+/HER2-, T2 to T4, N0 to N3, M0 breast cancer were randomized to receive anatrozole or fuvestrant for 4 to 6 months before surgery. Each center decided on adjuvant treatment according to the local policy. Patients with biopsy-proven BC underwent clinical, US and MRI examinations at baseline (M0), after 1 month of treatment (M1) and pre-operatively (Pre-op). Tumor response was defined on the longest tumor diameter on palpation, US and Dynamic Contrast-Enhanced (DCE)-MRI T1-weighted sagittal slices. All radiological and pathological data were submitted to centralized expert review. An optional tumor biopsy was performed at M1 to assess variation of Ki-67 index. Patients had no lymph node involvement on palpation, US and MRI at M0. Patients underwent surgery at 4 months when tumor response on palpation was insufficient (stable or progressive disease). In patients with partial clinical response, NET was continued for an additional 2 months. Adjuvant therapy after surgery was decided by a multidisciplinary board. The study was conducted according to the Declaration of Helsinki and Good Clinical Practice guidelines and all patients provided their written informed consent. The study was authorized by the French Health Authority and approved by the Ethics Committee (Ile de France VIII).

### FDG-PET/CT imaging

Patients underwent 3 serial FDG-PET/CT scans at M0, M1 and Pre-op in our institution from December 2007 to December 2010. Patients received an intravenous injection of 4–4.5 MBq/kg of 18-FDG in the arm opposite to the tumor, when capillary blood glucose level was less than 8 mmol/L and after fasting for at least 6 h. Whole body imaging PET/CT scans (from the vertex to mid-thighs) were acquired from 60 to 80 min after FDG injection on a PET/CT scanner (Discovery LS, General Electric Healthcare, Waukesha, WI, USA) on a 3D mode with 5–7 bed positions of 4–5 min each. Non contrast-enhanced CT images were acquired with the following parameters: 40 mAs, 140 kV, 5 mm section thickness, 0.8 s per CT rotation, 22.5 mm/s table speed. This acquisition was used for attenuation correction, fusion, and also for diagnosis. Immediately after the CT, PET data were collected in a caudo-cranial direction. The CT data were resized from a 512 × 512 matrix to a 128 × 128 one, in order to match the PET data and to fuse the images. Images were analyzed by two nuclear medicine physicians on a Xeleris workstation (General Electric HealthCare) with triangulation tools for 3D vision.

### Images analysis

SUVmax (Standard Uptake Value maximum) were measured by using a manually-delineated VOI (Volume of Interest) including the whole tumor. Early metabolic response was defined at M1 and late metabolic response was defined after at least 4 months of NET (Pre-op), in order to confirm the persistence of the metabolic response observed at M1. A cut-off of 40% for the SUVmax decrease (Delta-SUVmax) at M1 was used to differentiate 2 groups of patients: “metabolic responders” (mR) and “metabolic non-responders” (mNR), according to results of a previously published study [15]. It was defined using a ROC analysis (*p* = 0.006).

### Survival analysis

The correlation of early metabolic response with OS, RFS and PEPI score was studied. The PEPI score [16] combines pathological response (ypTN), Ki-67 index and ER Allred score and is relevant to predict RFS in NET setting [16].

### Statistical analysis

Unpaired and two-sided Student tests were used to confirm significant differences in Delta-SUVmax at M1 and at Pre-op between mR and mNR according to the 40% Delta-SUVmax cut-off, and to compare morphological response (palpation, US and MRI) and variation of Ki-67 index at each time-point (M0, M1 and Pre-op or surgery for Ki-67) in mR and mNR. A Fisher’s exact test was used to compare pathological response, PEPI score, BCS rate and adjuvant therapy (endocrine therapy or chemotherapy) between mR and mNR. Differences in OS and RFS were analyzed using the Kaplan-Meier method. The influence of histological type (ductal or lobular) and the endocrine treatment arm (anastrozole or fulvestrant) on SUVmax was also analyzed using a Student test. The *p* values considered as statistically significant were < 0.05 and most statistical analysis were done using GraphpadPrism7.0b and R softwares.

## Results

### Patient characteristics

**Fig. 2:**
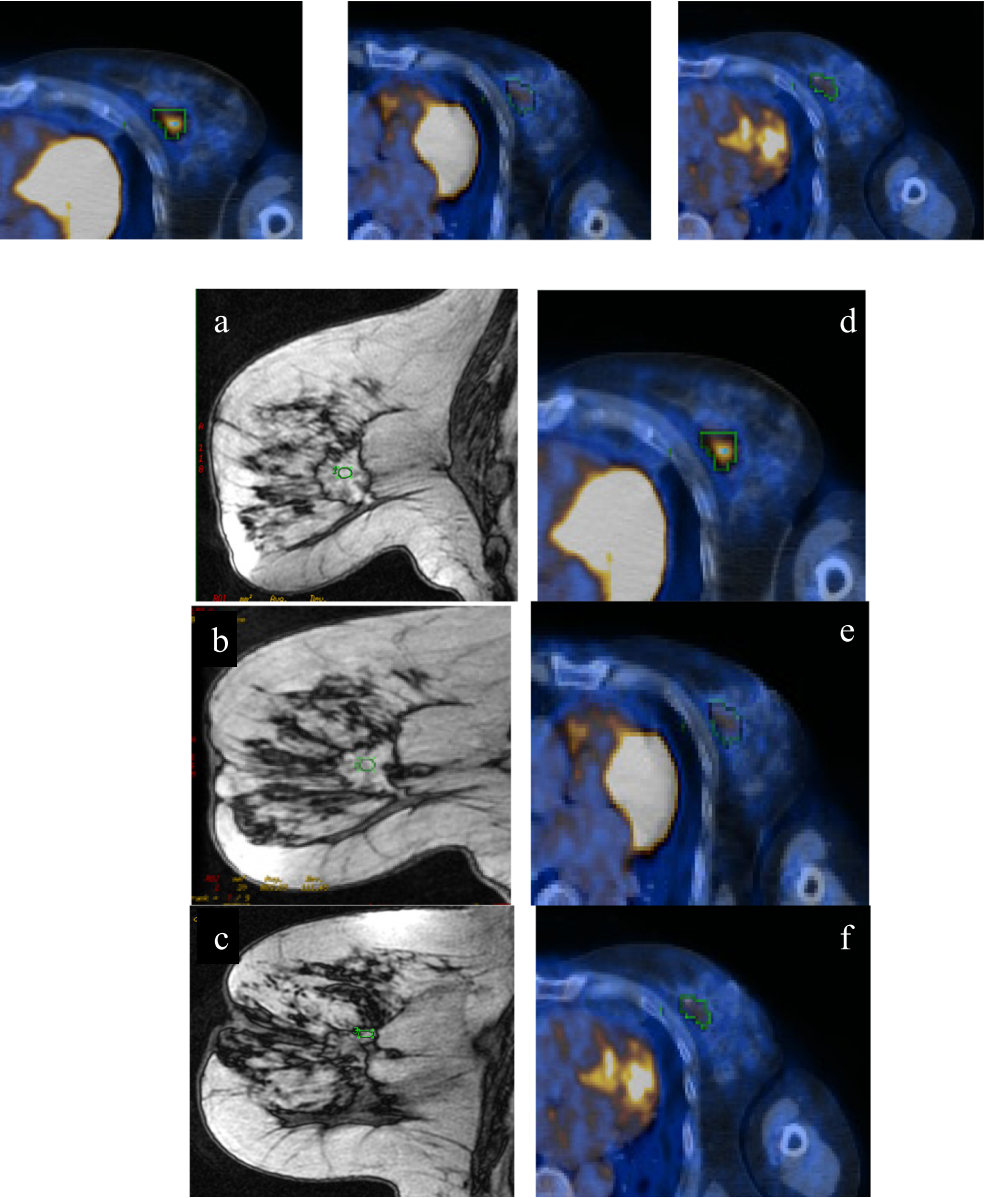
Decrease of tumor size on gadolinium-enhanced, T1-weighted sagittal MRI slices at M0 (**a**, 30 mm), M1 (**b**, 25 mm) and Pre-op (**c**, 22 mm) in a 76 year-old mR patient Metabolic response was demonstrated on FDG-PET/CT fused axial slices at M0 (**d**, SUVmax = 5), M1 (**e**, SUVmax = 3) and Pre-op (**f**, SUVmax = 3).

**Fig. 3:**
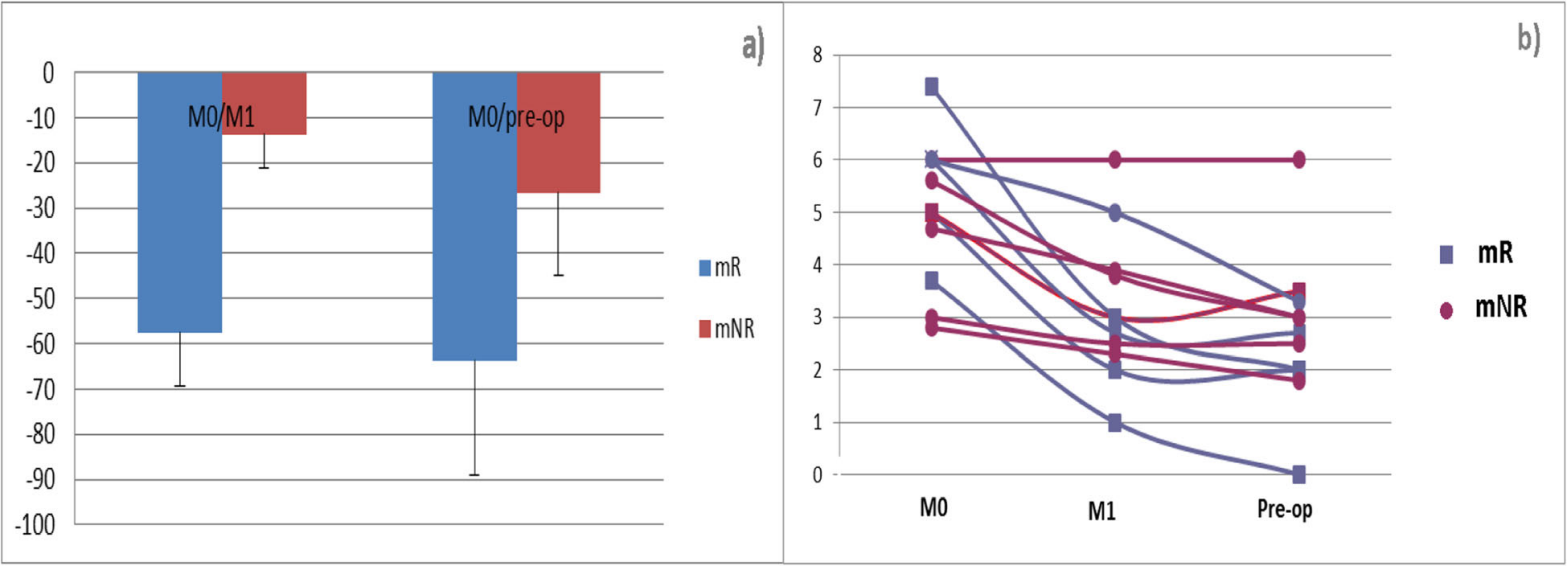
Variation of mean Delta-SUVmax in mR and mNR (**a**) and of absolute SUVmax in each patient (**b**)

Among the 116 patients enrolled in the CARMINA 02 trial between 2007 and 2011, 11 patients (mean age ± standard deviation: 74.2 y ± 3.6; range: 67–87 years) treated in our institution with 3 serial PET/CT scans available were included in the present study. All patients were clinically node negative. Patient baseline characteristics are presented in Table 1 and were comparable to those observed in the CARMINA 02 trial [14]. Seven of these 11 patients were randomized to anastrozole and 4 were randomized to fulvestrant. No significant differences in SUVmax according to treatment arm (*p* = 0.84, 0.74 and *p* = 0.71 at M0; M1 and Pre-op, respectively) or histological type (45% lobular vs 55% ductal carcinoma; *p* > 0.05) were observed, allowing global data analysis. Although tumor size was smaller in mR, no difference of SUVmax at M0 was found between mR and mNR (*p* = 0.41). According to the 40% Delta-SUVmax cut-off used at M1, 5 patients were classified as mR and 6 were classified as mNR (Figs. 2 and 3). Significant differences between mR and mNR were found at M1 (*p* = 0.0002) and Pre-op (*p* = 0.04) (Fig. 3).

**Table 1:** Baseline characteristics

Mean age ± SD (years)	74.2 ± 3.6
Mean tumor size; range (mm)	47 (30–70)
Tumor Stage	
T2	9
T3	2
Histological type	
Ductal	6
Lobular	5
Elston-Ellis Grade	
Grade I	1
Grade II	9
Grade III	1
Mean Ki-67 index (%)	12.4 ± 8.5
ER Allred Score	
6	1
7	2
8	8

### Morphological data

**Fig. 4:**
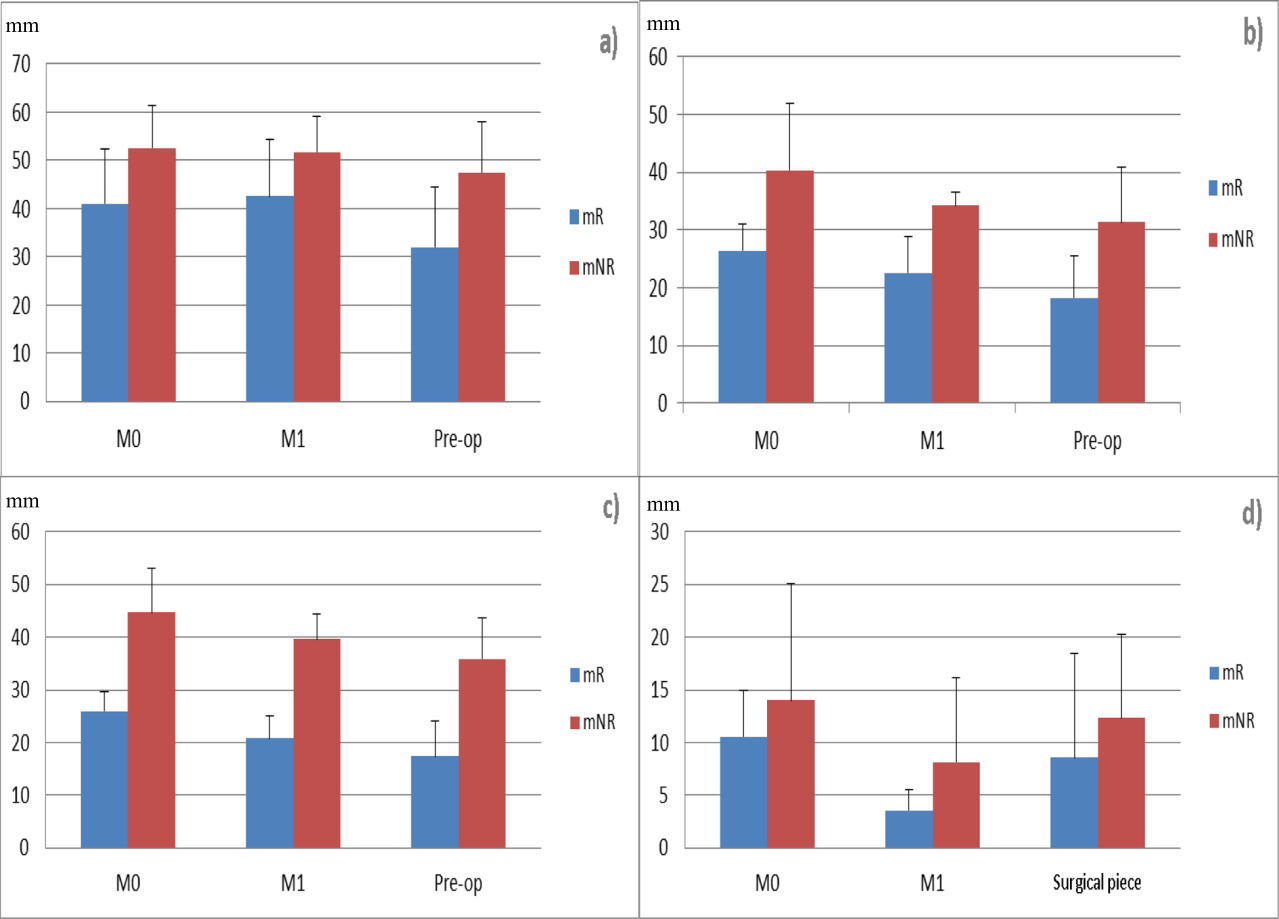
Variations of tumor size (mm) assessed on palpation (**a**), US (b), MRI (**c**) and of Ki-67 index (%) (**d**) at M0; M1 and Pre-op (a,b,c) or on surgical specimen (**d**) in mR and mNR

Morphological and pathological responses are summarized on Table 2. Tumor sizes on US or MRI were different between mR and mNR at M0, M1 and Pre-op, with the better *p*-value for MRI. Although not statistically significant, differences in the variation of tumor size between M0 and M1 were observed between mR and mNR [palpation (− 1% ± 12 vs − 0.2% ± 18.5; *p* = 0.9), US (− 15.4% ± 10.9 vs − 2.7% ± 22.8; *p* = 0.26) and MRI (− 20.5% ± 7.4 vs − 9.4% ± 16.8; *p* = 0.15), Fig. 4a,b,c]. Those differences persisted between M0 and Pre-op [palpation (− 23.7% ± 15.5 vs − 8.4% ± 21.7; *p* = 0.21), US (− 28.4% ± 22.2 vs − 20.5% ± 24.3; *p* = 0.6) and MRI (− 35.1% ± 20.7 vs − 18% ± 21.2; *p* = 0.21), Fig. 4a,b,c]. At Pre-op, a better clinical response was observed in mR: no progressive disease in mR vs 2 in mNR (*p* = 0.4) and 2 partial response in mR vs 1 partial response in mNR. Six patients were classified as having stable disease (*p* = 0.5). Two patients in each group had a partial response, as demonstrated by US or MRI and 7 patients were classified as having stable disease (*p* = 1).

**Table 2:** Metabolic, morphological and pathological measurements at M1, Pre-op and on the surgical specimen. Student tests were used to compare metabolic, morphological data and Ki-67 data in mR and mNR. Fisher’s exact tests were used for pathological data

		mR (5 pts)	mNR (6 pts)	*p* values
M0	**SUVmax (g/mL)**	5.4 ± 1.4	4.7 ± 1.5	0.41
	**Clinical size (mm)**	41 ± 11.4	52.5 ± 8.8	0.105
	**US size (mm)**	26.4 ± 4.7	40.3 ± 11.6	0.03
	**MRI size (mm)**	**26 ± 3.7**	**44.7 ± 8.3**	**0.0016**
	**Ki-67 (%)**	10.6 ± 4.4	14 ± 11.1	0.51
M1	**SUVmax (g/mL)**	**2.6** ± 1.1	**3.9** ± 1.4	0.00017
	**Clinical size (mm)**	42.5 ± 11.9	51.7 ± 7.5	0.23
	**US size (mm)**	**22.6 ± 6.3**	**34.2 ± 2.4**	**0.01**
	**MRI size (mm)**	**20.8 ± 4.2**	**39.7 ± 4.8**	**7.1 E-5**
	**Ki-67 (%)**	3.6 ± 1.9	8.2 ± 8	0.23
Pre-op	**SUVmax (g/mL)**	**2** ± 1.3	**3.3** ± 1.4	**0.018**
	**Clinical size (mm)**	32 ± 12.5	47.5 ± 10.4	0.06
	**US size (mm)**	**18.2 ± 7.3**	**31.3 ± 9.5**	**0.041**
	**MRI size (mm)**	**17.4 ± 6.7**	**35.8 ± 7.7**	**0.002**
BCS rate	**Number of patients**	2	2	1
Surgical specimen	**Ki 67 (%)**	8.6 ± 9.8	12.3 ± 7.9	0.41
	**Sataloff (TA + TB vs TC + TD) (n)**	1 vs 4	0 vs 6	1
	**PEPI score (I + II vs III) (n)**	4 vs 1	2 vs 4	0.24

### Pathological data

No significant difference in Ki-67 index was observed between mR and mNR at M0 (*p* = 0.51), M1 (*p* = 0.23) and on surgical specimen (*p* = 0.41) (Table 2; Fig. 4d). Although not significant, mR had a lower Ki-67 index at M0, M1 and on surgical specimen, and a higher decrease of the Ki-67 index at M1 (− 61.5% ± 20.9 vs + 29% ± 185; *p* = 0.21). No correlation between metabolic and pathological responses was found (Table 2), but pathological tumor size was significantly smaller in mR compared to mNR (22.8 mm ± 6.1 vs 42.5 mm ± 10.8; *p* = 0.0057). One patient with a complete metabolic response at Pre-op also presented the best pathological response (Sataloff TB), other patients were classified Sataloff TC (Table 2).

### Survival data

**Fig. 5:**
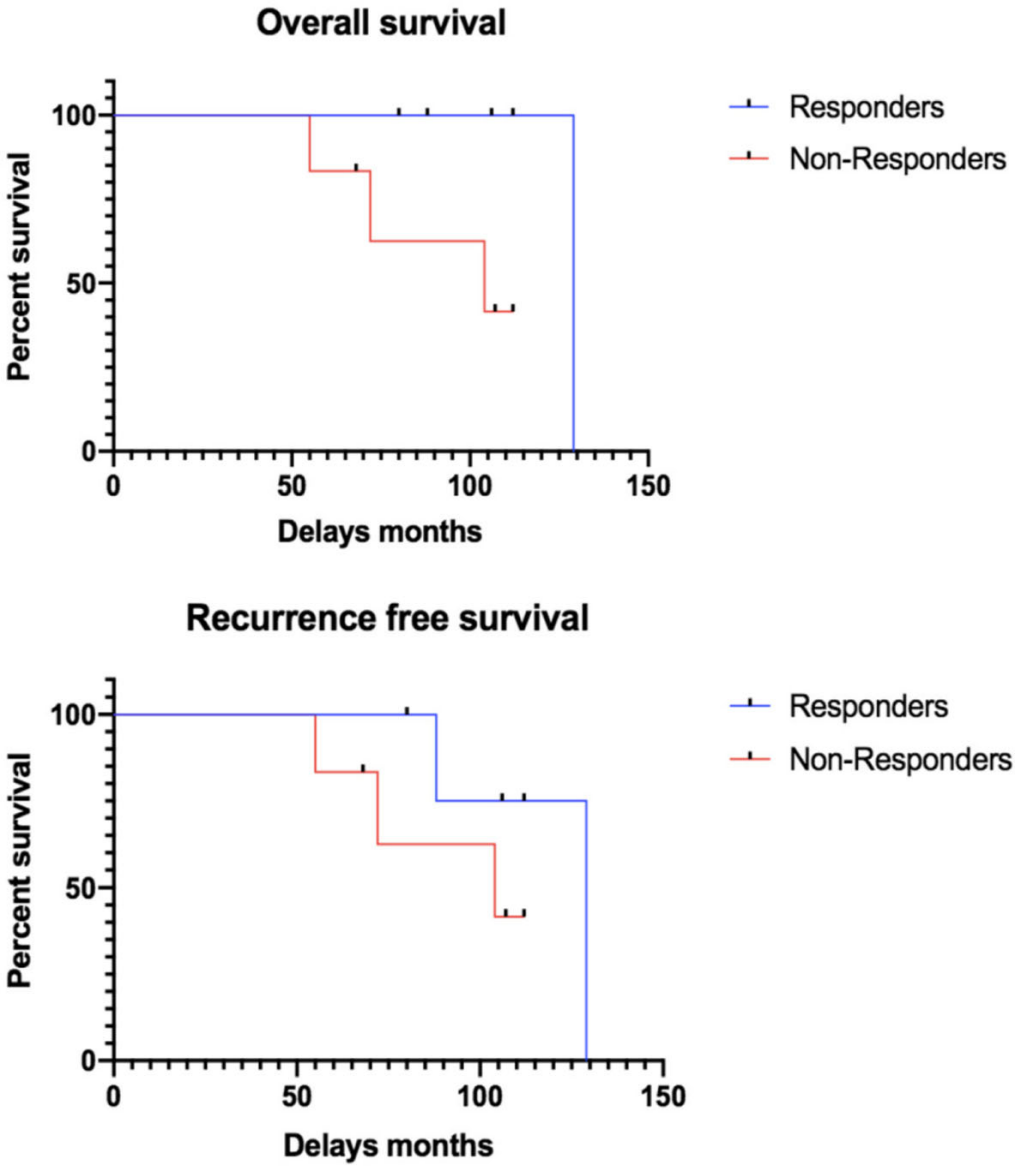
Survival parameters, overall survival (OS) and recurrence-free survival (RFS) in mR and mNR using Kaplan-Meier method

Mean follow-up was 93.8 ± 22.8 months. Three mNR died during follow-up: 1 from glioblastoma and 2 from disease progression out of 3 mNR with distant metastases (2 patients with bone metastases and 1 liver metastasis). mR had better OS and RFS, although not significant (Kaplan-Meier *p* = 0.08 and 0.06, respectively; Fig. 5). The PEPI score was different between mNR and mR, with a better prognostic index in mR (*p* = 0.24; Table 2). More mNR received adjuvant chemotherapy than mR (67% vs 20%; *p* = 0.24), because of a poor pathological response. No significant difference in BCS rate was observed between mR and mNR, with 2 patients in each group (*p* = 1).

## Discussion

Monitoring response to NET is challenging and no consensus has been reached concerning the best modality to use. Palpation and US are most commonly used in clinical practice, whereas breast MRI is increasingly used in clinical trials [7, 14]. Mammography is not a valuable tool because it underestimates tumor size compared to the surgical specimen [17]. FDG-PET/CT imaging has shown high performances in the NCT setting [18, 19]. Only one previously published study assessed the value of metabolic response to NET in 11 N0 BC patients with a high expression of ER (ER Allred score 7–8) [15]. Because we have previously shown the prognostic value of metabolic response to endocrine treatment on Progression Free Survival in metastatic BC patients [20], we decided to prospectively assess the value of metabolic response to NET in a homogenous population of postmenopausal women with non-metastatic, ER+/HER2- BC included in a clinical trial. Performances of FDG-PET/CT imaging were compared with those of morphological and pathological parameters. In the Ueda’s study [15], a 40% Delta-SUVmax cut-off was determined using a ROC analysis and after 4 weeks of NET. Therefore, with the same delay of 4 weeks between baseline PET (M0) and assessing PET (M1), we used the same Delta-SUVmax cut-off to define early metabolic response. This cut-off allowed us to differentiate mR and mNR with a significant difference at M1 that persisted at Pre-op. We were also able to compare metabolic response with morphological response and with Ki-67. However, compared to the Ueda’s study, in whom morphological imaging response was based on breast US alone, MRI data were incorporated in the morphological response criteria in the present study*.*

We found that patients with smaller tumors at diagnosis had a better pathological response in agreement with the literature [21]. mR had smaller tumors on morphological examinations, except on palpation which is known for poor reproducibility. In the present study, significant differences in tumor size decrease, assessed on US and MRI, were observed between mR and mNR at M1 and at Pre-op (Table 2), as well as in the Ueda’s study.

In the present study, although mNR had a higher Ki-67 index at each time-point compared to mR, no significant difference in variation of Ki-67 index was observed between mR and mNR at M1 or on surgical specimen. A wide range of values for Ki-67 index was also observed in both groups, likely related to the small sample size of our cohort, which may have prevented the demonstration of significant differences. In CARMINA 02 trial, Ki-67 index was significantly reduced from the first month with both treatments (anastrozole or fulvestrant) and its level at the time of surgery was associated with pathological, but not with clinical response [14]. Ueda et al. compared metabolic response to variation of Ki-67 index at 2 weeks and 12 weeks, date of surgery, and found significantly higher decreases of Ki-67 index at 2 weeks and on surgical specimen in mR compared to mNR [15]. Monitoring of Ki-67 index has been increasingly used in NET trials [5]. However, no consensus has been reached concerning neither the scoring method, the interpretation nor the standard cut-off of Ki-67 index [22]. The IMPACT trial has shown that variation of Ki-67 index, assessed after 2 weeks of NET, was more predictive of RFS than the baseline value [5]. pCR is the “gold standard” for assessment of response of BC to NCT, as it is correlated with prognosis [2]. However, pCR is rarely observed after NET [4, 14]. Moreover, there is no evidence at the present time that pCR constitutes a prognostic factor in NET setting in contrast with NCT. In the present study, no patient achieved pCR (Sataloff TA), but one patient with a complete metabolic response at Pre-op achieved the best pathological response (Sataloff TB). Similarly, Ueda et al. did not find any pCR [15].

In terms of survival, all cancer-related events, such as distant metastases and BC-related deaths, occurred in mNR and a trend towards better OS and RFS was observed in mR. A better prognosis according to PEPI score was observed in mR. It is noteworthy that, in the CARMINA 02 trial, PEPI score was the only variable significantly predictive of RFS [14].

The main limitation of the present study is the small number of patients included. According to Gebhart et al. [23], molecular imaging should be incorporated in translational research efforts. However, the present study illustrates the difficulty to include patients in imaging protocol in addition to a clinical trial. The fact that metabolic response based on FDG-PET/CT was an optional secondary endpoint in this trial was a critical limit and might explain that the rate of patients who underwent 3 PET/CT scans was as low as 10%. We were able to recruit in a single center 11 patients from 87 N0 patients among the 116 one enrolled in this multicenter, randomized trial carried out between October 2007 and April 2011. The patients’ age with a mean age of 74.2 y ± 3.6 was also a drawback for an easy recruitment to perform serial imaging exams.

Several limitations also concern the use of SUVmax in view of its marked variability and its failure to display tumor heterogeneity are well known but this parameter is widely used to monitor response of BC to NCT [19]. SUVmax has been shown to be useful in routine practice, as it is simple and reproducible and could be a valuable tool if FDG-PET/CT was validated in the NET setting [15]. Other semi-quantitative tools, such as Metabolic Tumor Volume, TLG (Total Lesion Glycolysis) or SUVpeak (average SUV value in a 10-voxel region including SUVmax) could be evaluated, although they have not been shown to be superior to SUVmax measurements and they have not been tested in monitoring response of BC to NET [24]. Tumor biopsies were generally performed after PET/CT scan. However, in 3 patients, they were performed before (1, 2 and 30 days, respectively). This action might generate inflammatory conditions which potentially induce increased FDG uptake and introduce a bias.

Almost one half of patients in this cohort presented with lobular carcinoma, which is more frequent in elderly patients [25], but no significant difference in SUVmax was observed according to histological type, probably due to the small number of patients. Glucose metabolism in lobular carcinoma has been shown to be lower than in ductal carcinoma [26] and should be taken into account in future studies together with the BC molecular subtype, which is also associated with variations in glucose metabolism [27]. The follow-up in this study could be considered to be relatively short. Longer follow-up is needed in this specific population of patients with low proliferative ER+/HER2- BC, who have a better prognosis than other BC molecular subtypes, but which may experience late relapses [28].

18F-FES (fluoro-estradiol) targets ER and visualizes its functional in vivo pathway, which could predict response of BC to therapy and guide therapy selection for each patient. ER expression is a prerequisite to initiate endocrine therapy, but does not accurately reflect activation of the ER pathway in the tumor. 18F-FES and 18F-FDG have been shown to be complementary tools to monitor response of ER+ BC to NET [29, 30]. However, most studies involving 18F-FES have been performed in metastatic settings and further investigations are needed to define the value of 18F-FES in the NET setting.

## Conclusion

Despite some limitations, this ancillary study of CARMINA 02 trial showed that early metabolic response can be more informative than morphological response and should be further investigated in a larger cohort of patients. If these results were confirmed, FDG-PET/CT could become a simple “surrogate marker” to monitor tumor response, especially as NET is a valuable treatment option in postmenopausal women with ER rich/HER2- BC. Assessment of early metabolic response allows adjustment of treatment, such as early “switch” to a more effective treatment option such as chemotherapy or targeted therapy, thereby improving patient care and prognosis.

## Data Availability

The clinical data belong to the Sponsor UNICANCER. The data are available and can only be uploaded to a platform that would be willing to sign a transfer agreement with Unicancer, in conformity with the European Commission’s Standard Contractual Clauses; In addition, data can only be accessed by experts that are identified in advance, to whom Unicancer should be able to grant a personal access.

## Abbreviations

BC: Breast cancer
BCS: Breast conserving surgery
ER: Estrogen receptor
FDG: 2-deoxy-2-[18F]fluoro-D-glucose
mNR: Metabolic non-responders
mR: Early metabolic responders
MRI: Magnetic resonance imaging
NCT: Neoadjuvant chemotherapy
NET: Neoadjuvant endocrine therapy
OS: Overall survival
pCR: Pathological complete response
PEPI: Preoperative endocrine prognostic index
PET/CT: Positron emission tomography/computed tomography
Pre-op: Pre-operatively
RFS: Recurrence-free survival
SUVmax: Standard uptake value maximum
US: Breast ultrasound
